# Zero-contrast imaging for the assessment of transcatheter aortic valve implantation in candidates with renal dysfunction

**DOI:** 10.1080/0886022X.2023.2224888

**Published:** 2023-06-23

**Authors:** Guy F. A. Prado, Stefano Garzon, Jose Mariani, Adriano Caixeta, Breno O. Almeida, Felipe O. Ramalho, Marcelo L. C. Vieira, Claudio H. Fischer, Gilberto Szarf, Walther Ishikawa, Pedro A. Lemos

**Affiliations:** a Department of Interventional Cardiology, Hospital Israelita Albert Einstein, São Paulo, Brazil; b Department of Echocardiography, Hospital Israelita Albert Einstein, São Paulo, Brazil; c Department of Radiology, Hospital Israelita Albert Einstein, São Paulo, Brazil

**Keywords:** Aortic stenosis, transcatheter aortic valve implantation, renal failure, contrast-induced nephropathy

## Abstract

**Background:**

Candidates for transcatheter aortic valve implantation (TAVI) are currently evaluated using computed tomography angiography and invasive cardiac catheterization as an essential part of case selection and pre-procedure interventional planning. However, both imaging methods utilize iodinated agents, which may cause contrast-induced nephropathy, particularly in patients with baseline renal dysfunction. This study aimed to describe a zero-contrast imaging protocol for pre-TAVI evaluation in patients with advanced renal impairment.

**Methods:**

The pre-TAVI zero-contrast scheme consisted of the following multi-modality combinations: (1) gadolinium-free magnetic resonance imaging (three-dimensional navigator-echo with electrocardiogram-gated steady-state free-precession series); (2) iodinated-free multislice computed tomography electrocardiogram-gated; (3) lower limb arterial duplex scan ultrasound; and (4) transesophageal echocardiography. Ultimately, TAVI was performed for those deemed good candidates, and contrast was allowed during the intervention; however, operators were strongly advised to utilize the least volume possible of iodinated agents. This pilot survey included ten patients with symptomatic aortic stenosis and renal dysfunction who underwent zero-contrast multi-modality imaging.

**Results:**

All the patients ultimately underwent TAVI. The intervention was successful in all cases, without ≥ moderate residual aortic regurgitation, prosthesis embolization, annulus rupture, major vascular complications, stroke, or death during index hospitalization. The creatinine clearance remained stable throughout the observation period (baseline: 26.85 ± 12.55 mL/min; after multi-modality imaging: 26.76 ± 11.51 mL/min; post-TAVI at discharge: 29.84 ± 13.98 mL/min; *p* = 0.3 all).

**Conclusion:**

The proposed contrast-free imaging protocol appears to be a promising clinical tool for pre-TAVI evaluation in patients with severe renal dysfunction.

## Introduction

Pre-procedure planning is key to avoiding complications and improving outcomes in patients undergoing transcatheter aortic valve implantation (TAVI) [1]. Currently, operators perform TAVI only after carefully evaluating the size of the aortic annulus, status of vascular routes, degree and distribution of calcific deposits in the valve apparatus, and height of the coronary ostia, among other features, in an attempt to optimize the procedural strategy. Mostly, this assessment is based on imaging modalities that include invasive coronary angiography [2] and multislice computed tomography angiography (CTA) [3], which are universally considered mandatory components of the pre-TAVI routine for most, if not all, patients. However, both diagnostic methods use iodinated contrast agents, which may be a limitation in this population.

Chronic renal dysfunction is common in individuals with aortic stenosis and is often challenging for the evaluation of TAVI candidates [4–6]. It is widely acknowledged that the use of contrast agents can result in a deterioration of renal function, particularly in certain subsets of clinical scenarios. Despite the widespread use of angiographic contrast-based methods in pre-TAVI evaluations, the occurrence of acute kidney injury (AKI) in this specific context has been underreported [7,8]. Nonetheless, everyday practice and common sense indicate that it is a significant clinical concern that warrants further attention.

In this context, we developed a zero-contrast diagnostic routine for patients with severe aortic stenosis and renal dysfunction who are considered for TAVI. This report summarizes the proposed imaging scheme and the results of its application in an initial series of cases.

## Methods

### Study population and definitions

This pilot survey comprised a population of all patients with severe symptomatic aortic stenosis [9] and renal impairment, defined as an estimated creatinine clearance [10] of < 60 mL/min, who underwent pre-TAVI zero-contrast imaging evaluation at our institution.

The Valve Academic Research Consoritum 3 (VARC-3) definitions were used for procedure success, vascular access site, access-related complications, cerebrovascular events, procedure-related myocardial infarction and bleeding [11]. VARC-3 recommends using the widely recognized Kidney Disease: Improving Global Outcomes (KDIGO) definition of acute kidney injury [12]. The study was conducted in accordance with the principles of the Helsinki Declaration and was approved by the institutional review board and the ethics committee of the Albert Einstein Hospital (CAAE: 55965922.3.0000.0071; approved on 26 February 2022), which waived the need to obtain consent for the collection, analysis, and publication of this retrospective, non-interventional, series of cases.

### Pre-TAVR multi-modality imaging protocol

The imaging routine was developed to fulfill the following premises:

1. Imaging had to be free of iodine- and gadolinium-based contrast agents;
2. Assessment had to be noninvasive;
3. The combination of multi-modality imaging needed to provide information on:

- Vascular access and femoral-iliac-aortic anatomy (dimensions, presence of atherosclerosis or calcification, tortuosity);
- Aortic annulus sizing;
- Left ventricle outflow tract, aortic valve, and aortic calcium distribution;
- Angulation between the left ventricle and the aorta;
- Calcification pattern and patency of proximal coronaries, as well as coronary ostia heights.

The protocol comprised a multi-modality imaging ­combination that included: (1) gadolinium-free magnetic resonance imaging (MRI) (three-dimensional navigator-echo with steady-state free-precession series and electrocardiogram-gated), (2) iodinated-free multislice computed tomography electrocardiogram (ECG)-gated, (3) lower limb arterial duplex scan ultrasound, and (4) transesophageal echocardiogram. All patients underwent all four categories of free-contrast imaging examinations, and the results were assessed only for those of satisfactory quality. The imaging examinations were conducted either before or during the hospitalization for the index TAVI procedure, as part of the proposed protocol. Contrast was allowed during the intervention for those deemed good candidates for TAVI. Prior to the interventional valve procedure, creatinine levels were measured for all ten patients both before and on the day following the contrast-free imaging examinations. Subsequent to the TAVI procedure, creatinine levels were collected daily for a minimum of 72 h or for a longer duration if clinically indicated. As part of the discharge process, a final creatinine sample was obtained for control purposes.

**Figure 1.**
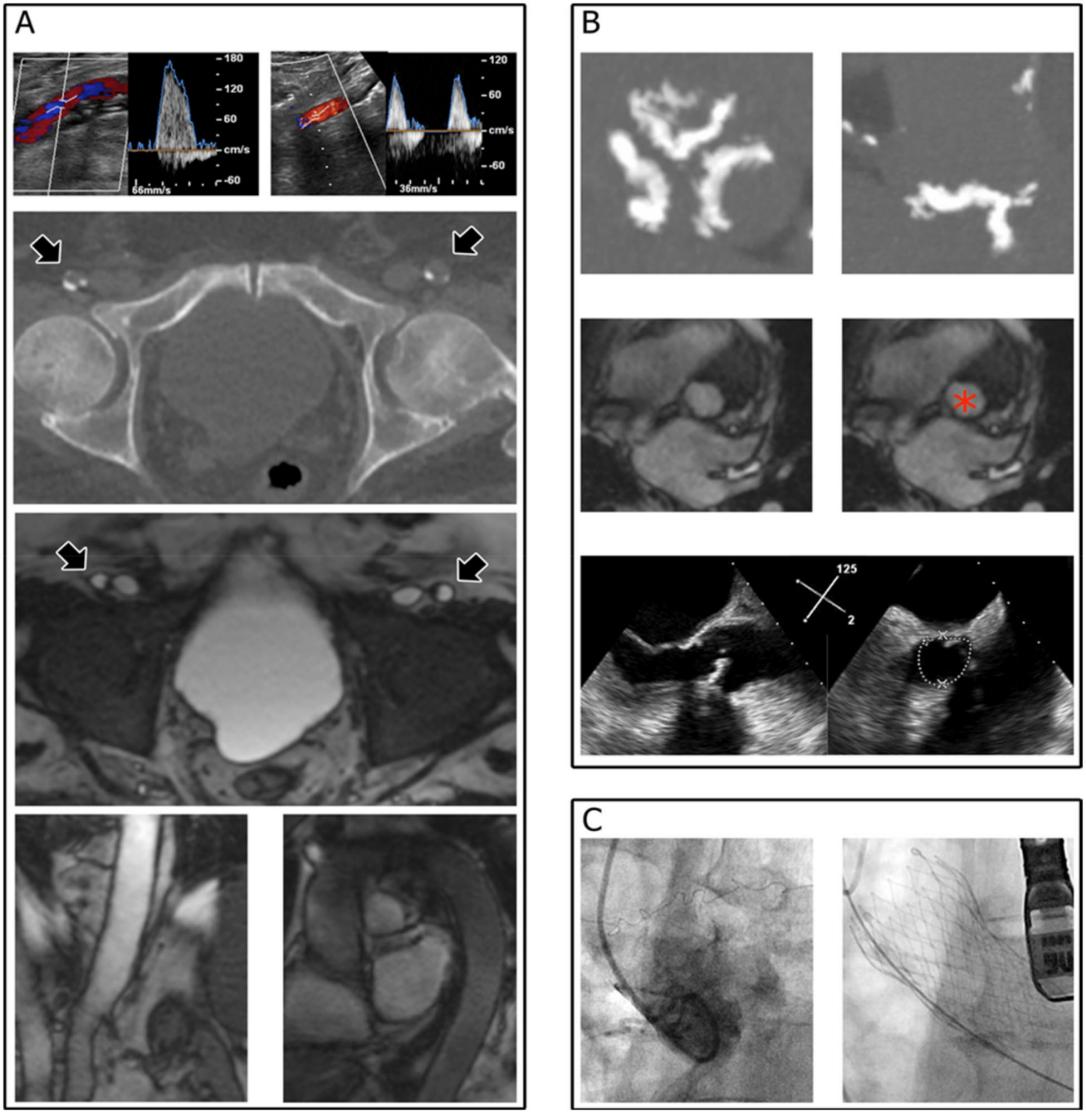
Multimodality imaging for the assessment of TAVI candidates with renal dysfunction. Femoro-iliac-aortic endovascular access (A) was evaluated by duplex scan ultrasound (upper panel showing the left and right femoral arteries), computed tomography (arrowheads in the mid-upper panel indicate the left and right femoral arteries), and nuclear magnetic resonance imaging (arrowheads in the mid-upper panel indicate the left and right femoral arteries). The aortic root and aortic annulus (B) were evaluated using computed tomography (upper panel), nuclear magnetic resonance imaging (red asterisk in the middle panel indicates the aortic annulus region), and echocardiography (lower panel), which were reassessed for confirmation during the procedure (C, left panel). The final shape of the metallic frame of the prosthesis is shown in the right panel of panel C.

Magnetic resonance imaging was performed with three-dimensional gradient echo and steady-state free precession cine images to evaluate the heart, aorta, iliac, and femoral vessels [13]. Computed tomography was performed using a 320-row multislice scanner (Aquilion One, Canon Medical System Corporation, Tokyo, Japan) with variable pitch to evaluate the heart and thoracic aorta with retrospective ECG gating and the abdominal aorta and iliac and common femoral arteries without ECG synchronization [13]. The aorta and lower limb arteries were also assessed using duplex ultrasound. Transthoracic and/or transesophageal echocardiography, preferably with tridimensional imaging, was performed in all patients. Additionally, transesophageal echocardiography was performed prior to any manipulation. The findings from all modalities were combined (Figure 1) to determine the final therapeutic strategy. Ultimately, TAVI was indicated for those deemed to be good candidates by our institutional Heart Team. Restricted administration of iodinated contrast was allowed during the intervention to re-assess cardiac, coronary, and vascular anatomy, but operators were strongly advised to utilize the least possible volume of iodinated agents.

### Statistical analyses

Categorical variables are presented as numbers (percentages), and continuous variables are presented as mean ± standard deviation for normally distributed data or median and interquartile range for non-normally distributed data. To compare creatinine clearance groups (baseline, post imaging, and post-TAVI), we used a general linear model test. We performed a paired sample t-test to compare baseline and post-TAVI creatinine clearance. Differences were considered statistically significant at two-tailed *p*-values < 0.05. All analyses were performed using SPSS version 25 software (IBM Corp., Armonk, NY, USA).

## Results

Between July 2017 and June 2022, 188 patients underwent TAVI at our institution. The proposed zero-contrast pre-TAVI evaluation was applied to 10 male patients. The mean age was 81.26 (±12.2), diabetes was present in 7 patients; the mean Society of Thoracic Surgeons score for mortality was 10.9 (±8.7), the median aortic valve calcium score was 2709.5 AU (IQR: 1601.75–4218.25). At baseline, mean serum creatinine and creatinine clearance were 2.64 ± 0.72 mg/dL and 26.85 ± 12.55 mL/min, respectively. In seven patients, the aortic annulus area was observed to be numerically larger when evaluated using MRI as opposed to TEE. Among these patients, a size difference of more than 10% was evident in five cases, when comparing both assessment methods. Conversely, in the remaining three patients, TEE demonstrated a larger annulus size than MRI. However, among these cases, the differences were less than 2% in two instances, with only one case showing a difference of 9.5%. The clinical characteristics and main findings of the multi-modality imaging are summarized in Tables 1 and 2, respectively. The renal function remained unaltered after the multi-modality imaging protocol (post imaging serum creatinine: 2.66 ± 0.73 mg/dL; post imaging creatinine clearance: 26.76 ± 11.51 mL/min; *p*-value = 0.8 and 0.9 respectively). After TAVI, there was a numerically slight improvement in renal function compared to baseline, but without statistical significance (post-TAVI serum creatinine: 2.45 ± 0.89 mg/dL; post-TAVI creatinine clearance: 29.84 ± 13.98 mL/min; *p*-value = 0.4 and 0.1 respectively).

**Table 1.** Patients characteristics.

	Patient population (*n* = 10)
Age, years	81.26 (±12.2)
Male, n (%)	10 (100)
Body mass index, Kg/m^2^	27.0 (±4.2)
Diabetes n (%)	7 (70.0)
Heart failure NYHA^§^ class III or IV (%)	10 (100)
STS^ll^ score, %	10.9 (±8.7)
Baseline creatinine clearance, mL/min	26.8 (±12.5)
Echo LV^‡^ ejection fraction, %	45.7 (±13.3)
Aortic valve area, cm²	0.81 (±0.20)
Mean pressure gradient, mmHg	33.2 (±10.4)
CT ^†^Aortic valve calcium score, AU*	2709.5 (1601.75–4218.25)

Numbers are means (± standard deviations), medians (interquartile ranges), or counts (percentages).

*AU = Agatston units; ^†^CT = computed tomography; ^‡^Echo–LVEF = left ventricular ejection fraction determined by echocardiography; ^§^NYHA = New York Heart Association; ^ll^STS = Society of Thoracic Surgeons.

**Table 2.** Main findings of multi-modality imaging.

	Patient #1	Patient #2	Patient #3	Patient #4	Patient #5	Patient #6	Patient #7	Patient #8	Patient #9	Patient #10
Transesophageal echocardiogram										
Aortic annulus area, mm²	549	554	314	663	443	372	544	415	460	427
Left ventricular ejection fraction, %	31	46	59	30	52	25	61	55	40	58
Mean pressure gradient, mmHg	27^a^	16^a^	41	34^a^	53	25^a^	43	28^b^	31	34
Magnetic resonance										
Sinus of Valsalva, mm	35	35.5	30.5	38	32	36.7	34.3	41	40.6	33
Aortic annulus perimeter, mm	83	94	70	93	83	85	84	77	84	72
Aortic annulus area, mm²	542	630	385	683	519	506	535	421	487	390
Femoral-iliac smallest diameter, mm	–	9	7	11	–	–	–	10	9	–
Lowest coronary ostium, mm	16	–	8	12	10	10	13	17	11	–
Computed tomography										
Aortic valve calcium score, AU	1248	1565	1772	4399	4158	3946	3647	1614	5767	1628
Left ventricular outflow tract calcification	Yes	No	Yes	Yes	No	Yes	No	No	yes	No
Femoral-iliac smallest external diameter, mm	5	7	7	9	8	–	9	10	11	9
Lowest coronary ostium, mm	13	14	11	12	12	12	14	16	12	12
Severe calcification in proximal LCA*	Yes^c^	Yes	No	No	Yes	Yes	No	Yes	No	Yes
Severe calcification in proximal RCA^†^	Yes^c^	No	No	Yes	Yes	Yes	Yes	Yes	Yes	Yes
Lower limbs artery ultrasound										
Stenosis > 50%	No	No	No	–	No	No	–	Yes	No	Yes

*LCA, left coronary artery; ^†^RCA, right coronary artery.

^a^Low-gradient/low flow.

^b^Paradoxical low-gradient severe aortic stenosis.

^c^Visible patent left internal mammary artery graft to left anterior descending artery.

All procedures were carried out *via* transfemoral access, using either balloon-expandable (*n* = 7) or self-expandable (*n* = 3) prostheses. The median volume of contrast administered during the procedures was 42.5 mL (interquartile range: 35.0–63.7). No device embolization or severe residual aortic regurgitation was observed. In one case, mild-to-moderate final aortic regurgitation was observed after implantation of a well-positioned 31-mm self-expandable valve, related to massive and asymmetric aortic valve calcification. In addition, there were no cases of annulus rupture, patient-prosthesis mismatch, or in-hospital vascular complications. One patient required dialysis after the procedure due to refractory hypervolemia, and two patients underwent definitive cardiac pacemaker implantation. All the patients were discharged to their homes. Details of the procedure and in-hospital outcomes are presented in Tables 3 and 4, respectively.

**Table 3.** Procedure characteristics.

	Patient #1	Patient #2	Patient #3	Patient #4	Patient #5	Patient #6	Patient #7	Patient #8	Patient #9	Patient #10
Procedural characteristics										
Transfemoral access	Yes	Yes	Yes	Yes	Yes	Yes	Yes	Yes	Yes	Yes
Prosthesis type	Sapien 3	Sapien 3	Evolut R	Corevalve	Evolut R	Sapien 3	Sapien 3	Sapien 3	Sapien 3	Sapien 3
Prosthesis size, mm	29	29	26	31	29	26	29	26	26	26
Valve embolization	No	No	No	No	No	No	No	No	No	No
Residual mean aortic gradient, mmHg	6	5	8	15	9	4	7	9	5	4
Residual aortic regurgitation	Trivial	Trivial	Mild	Mild-to moderate	Mild	Mild	None	None	Trivial	None
Volume of contrast, mL	40	160	77	60	40	45	35	35	33	45
Need for percutaneous coronary intervention	‘ No	No	No	No	Yes	Yes	No	No	No	No

**Table 4.** Renal function and in-hospital clinical outcomes.

	Patient #1	Patient #2	Patient #3	Patient #4	Patient #5	Patient #6	Patient #7	Patient #8	Patient #9	Patient #10
Death	No	No	No	No	No	No	No	No	No	No
Hospitalization length, days	4	13	3	4	4	18	6	12	4	5
Vascular complications	No	No	No	No	No	No	No	No	No	No
Bleeding complications	No	No	No	No	No	No	No	Yes^a^	No	No
Renal Function										
Baseline serum creatinine, md/dL	2.01	3.10	2.39	3.16	2.33	3.2	1.83	3.3	3.65	1.49
Baseline creatinine clearance, mL/min	50.19	18.32	23.72	41.10	24.62	17.49	26.9	18.03	9.91	38.26
Post imaging serum creatinine, md/dL	2.26	3.01	2.37	3.08	2.45	2.93	1.73	3.25	3.98	1.56
Post imaging creatinine clearance, mL/min	44.64	18.87	23.92	42.16	23.41	19.16	31.49	18.36	9.16	36.54
Post-TAVI* (at discharge) serum creatinine, mg/dL	2.24	4.00	2.27	2.37	1.71	2.34	1.62	4.01	2.54	1.46
Post-TAVI*(at discharge) creatinine clearance, mL/min	45.04	14.2	24.97	55.26	33.54	23.91	33.44	14.84	14.24	39.04
Need for dialysis	No	Yes^b^	No	No	No	No	No	No	No	No

*TAVI = Transcatheter aortic valve implantation.

^a^Gastrointestinal bleeding associated with *Clostridium difficile* infection; VARC-3 type 1 of bleeding [12].

^b^Ultrafiltration modality due to congestive symptoms after transcatheter aortic valve implantation.

## Discussion

The main objective of the present report was to describe a comprehensive zero-contrast imaging protocol to evaluate candidates for TAVI in a group of patients with moderate-severe renal dysfunction. The scheme appeared to be feasible and safe in an initial series of ten cases, as cardiac and vascular morphologies were adequately defined, allowing for successful and uneventful interventional procedures.

The precise measurement of the aortic annulus is crucial for accurate prosthesis sizing prior TAVI. Typically, ECG-gated CTA has been the preferred method for this assessment. However, in our study, we opted to evaluate the aortic annulus dimensions solely using gadolinium-free MRI and TEE. Despite observing discrepancies between these two methods, with MRI indicating a numerically larger aortic annulus area in seven patients and TEE showing a larger area in three patients, the operators appeared to have appropriately chosen the correct prosthesis size, as no procedural complications or suboptimal results were encountered. Significantly, in the seven cases where measurements approached borderline values, the larger prosthesis size, which aligned more closely with the MRI findings, was chosen. Among the remaining three cases, only one (patient 10) exhibited a 9.5% discrepancy, with TEE indicating a larger aortic annulus compared to MRI, while the other two cases demonstrated differences of less than 2%. This observation raises the possibility of considering a minimalist procedure, foregoing TEE and general anesthesia. Previous studies have highlighted the tendency of TEE to underestimate the aortic annulus size in comparison to CTA [14,15]. Furthermore, strong correlations have been established between gadolinium-free MRI and CTA for aortic annulus sizing [16,17]. Another beneficial aspect of our decision-making process regarding prosthesis size and vascular access was the minimal use of contrast media, limited solely to the procedure itself.

Assessment of valvular calcification in the left outflow tract can be challenging using MRI [13]. To overcome this difficulty, we used a no-contrast CT scan, which allowed for easy identification of calcification. It is noteworthy that the only instance of paravalvular leak beyond mild was observed in an older generation of a self-expandable prosthesis in association with massive and asymmetric aortic valve calcification. We are of the opinion that the utilization of contrast-enhanced CT would not have impacted our valve type or size selection. The non-contrast CT scan accurately identified substantial calcification on the valve plane and in the left ventricular outflow tract, which led to the choice of a self-expandable prosthesis over a balloon-expandable one to minimize the risk of annular rupture. The MRI and TEE accurately determined the annulus size, and the largest available valve was selected. Nonetheless, the self-expandable prosthesis that was available at that time was an older generation without a skirt-sealing device, which has been demonstrated to reduce paravalvular leaks in newer generations [18]. Combining imaging methods is also helpful for evaluating vascular access. MRI, CT, and duplex Doppler allowed the assessment of peripheral artery sizes, as well as the presence of disease, tortuosity, and calcification. All the patients were treated using transfemoral access, and no vascular complications were observed.

A large proportion of patients who undergo TAVI have renal dysfunction and multiple comorbidities, which could predispose them to AKI after the procedure. In fact, up to 35% of patients develop AKI following TAVI, which is associated with increased in-hospital and 1-year mortalities [5,19]. Curiously, however, a sizable number of patients with aortic stenosis and kidney impairment at baseline can even have improved renal function after TAVI, indicating that the procedure may have a protective effect [4]. This context led us to allow minimum contrast utilization during, and only during, the procedure, mainly aimed at refining the positioning of the prosthesis. The median contrast volume used in our study was 42.5 mL (interquartile range: 35.0–63.7) which was numerically lower than the historical series [20,21]. It is important to highlight that as zero contrast was used prior to the intervention, no cumulative nephrotoxic effect was expected for the contrast used during treatment. Indeed, renal function persisted stable after the procedure in the majority of patients. Another important aspect to mention is that this represents the initial experience of our center. With the improvement of the operators’ learning curve in performing this strategy, we may be able to enhance our protocol and use less contrast volume in future cases or even consider eliminating it altogether, as reported by other centers with excellent results [22].

The prevalence of coronary artery disease is high in TAVI candidates [23] and is frequently found in pre-operative [24] assessment using coronary angiography or coronary CTA in patients frequently undergoing PCI before TAVI. However, a revascularization strategy can be challenging in this setting. The symptoms of both pathologies are similar, and there is a scarcity of data indicating that preemptive PCI in these patients can improve outcomes [25]. In our report, we only proceeded to coronary angiography at the same time as TAVI in selected patients, and PCI was performed only if critical proximal lesions were found or if future difficult coronary reaccess was considered a possibility. The two cases in which we performed PCI involved important, calcified long lesions that could not be fully predicted despite pre-dilation. Sometimes it is necessary to use techniques such as rotational atherectomy, lithotripsy, or a combination of strategies to successfully fracture the calcium a condition that can be challenging after the valve implantation. In addition, one of the cases involved the treatment of the ostium of the right coronary artery, while the other required treatment of a bifurcation lesion in the left descending artery/diagonal branch. As previously shown by our group, intravascular ultrasound-guided PCI is a helpful tool to save contrast, and was also applied in the current study population when needed [26]. Importantly, there were no cases of in-hospital myocardial infarction following TAVI in our study.

Our study has obvious limitations that hinder the extrapolation of the results. Firstly, it was conducted as a retrospective single-arm design, characterized by a small sample size and limited follow-up. Consequently, the presence of selection bias cannot be ruled out, and the positive findings observed in this series of 10 cases necessitate cautious interpretation. Still, despite the absence of a control group in this report, it is worth noting that all the procedures were successfully conducted according to the VARC-3 definitions. Second, MRI was compared with TEE, a method known to underestimate annulus measurements. Third, all the echo exams for the assessment of the vascular route and the measurements of the aortic valvular plane were performed by experienced operators what hinders to expand to every center. In our view, only highly experienced operators in high-volume cardiovascular centers should conduct these complex procedures. This ensures that patients receive the best possible care and minimizes the intrinsic risks associated with these procedures. Finally, with the widespread adoption of TAVI in various clinical scenarios, including bicuspid valves, it is well-known that the evaluation of anatomy and selection and delivery of the prosthesis can be challenging, thus potentially limiting the protocol. It is noteworthy that none of the ten patients in our study had bicuspid valve anatomy. The sole intent of this study was to present our initial experience with TAVI planning using a contrast-free protocol and the results. This seems to be a promising clinical tool for pre-TAVI evaluation of patients with severe renal dysfunction.

## Conclusions

In patients with severe renal dysfunction, the novel contrast-free multi-modality imaging scheme was shown to be feasible with appropriate results for patient candidates for TAVI.
